# Dissociation between Semantic Representations for Motion and Action Verbs: Evidence from Patients with Left Hemisphere Lesions

**DOI:** 10.3389/fnhum.2017.00035

**Published:** 2017-02-14

**Authors:** Lawrence J. Taylor, Carys Evans, Joanna Greer, Carl Senior, Kenny R. Coventry, Magdalena Ietswaart

**Affiliations:** ^1^Department of Psychology, Northumbria UniversityNewcastle upon Tyne, UK; ^2^Department of Psychology, Goldsmiths University of LondonLondon, UK; ^3^Department of Psychology, School of Life and Health Sciences, Aston UniversityBirmingham, UK; ^4^School of Psychology, University of East AngliaNorwich, UK; ^5^Department of Psychology, University of StirlingStirling, UK

**Keywords:** neuropsychology, left hemisphere, lateral occipitotemporal cortex, affordances, embodied cognition, semantic representation, aphasia

## Abstract

This multiple single case study contrasted left hemisphere stroke patients (*N* = 6) to healthy age-matched control participants (*N* = 15) on their understanding of action (e.g., holding, clenching) and motion verbs (e.g., crumbling, flowing). The tasks required participants to correctly identify the matching verb or associated picture. Dissociations on action and motion verb content depending on lesion site were expected. As predicted for verbs containing an action and/or motion content, modified *t*-tests confirmed selective deficits in processing motion verbs in patients with lesions involving posterior parietal and lateral occipitotemporal cortex. In contrast, deficits in verbs describing motionless actions were found in patients with more anterior lesions sparing posterior parietal and lateral occipitotemporal cortex. These findings support the hypotheses that semantic representations for action and motion are behaviorally and neuro-anatomically dissociable. The findings clarify the differential and critical role of perceptual and motor regions in processing modality-specific semantic knowledge as opposed to a supportive but not necessary role. We contextualize these results within theories from both cognitive psychology and cognitive neuroscience that make claims over the role of sensory and motor information in semantic representation.

## Introduction

The motor system is primarily engaged for the execution of actions, but has also been shown to be engaged when familiar actions are observed (e.g., Calvo-Merino et al., 2005), imagined (e.g., Decety, 1996), or even read about (Beilock et al., 2008). For example, reading a sentence describing an action sometimes primes bodily movements matching the referential content (e.g., Glenberg and Kaschak, 2002; Papesh, 2015). Such evidence is frequently taken to support the notion that bodily and action representations are routinely recruited to derive meaning from language (Gallese and Lakoff, 2005; Fischer and Zwaan, 2008). Research over the past decade has demonstrated that language describing familiar actions results in activation of motor systems (e.g., Pulvermuller, 2005; Kemmerer et al., 2008). However, despite the broad and high-profile theoretical claims made in the literature about language understanding and sensorimotor systems, the necessity of such recruitment has not been firmly established. For example, the effects found might be merely epiphenomenal or the case may be that “sensory and motor information plays, at best, a supportive but not necessary role in representing concepts” (Mahon and Caramazza, 2008, p. 67). This debate has led others to propose a middle ground—that relying on both “embodied” and “symbolic” mechanisms provides language users with richer and more fault-tolerant representations (Andrews et al., 2009; Dove, 2009; Taylor and Zwaan, 2013). What would clarify this debate however, is evidence to suggest that “embodied” and “symbolic” representations dissociate, and also that varying “perceptual” brain regions may be implicated even within a semantic category. Indeed verbs do not always refer to concrete, dynamic actions; verbs can also refer to events involving movement, mental states, and can state a change. A raindrop might fall to earth and a flower might wilt, resulting in visual motion, but we cannot directly realize such events with our bodies as we might when we hit (a concrete, dynamic action; as described in Table 1 labeled +Action/+Motion verbs) or hold an object (a motionless action; as described in Table 1 labeled as the +Action/−Motion Category in our research design).

**Table 1:** **Example linguistic stimuli**.

	**+Motion**	**−Motion**
+**Action**	**I. Concrete, dynamic actions** throwing, chopping	**II. Motionless actions** holding, ogling
−**Action**	**III. Observable events** crumbling, flowing	**IV. Mental states** hoping, desiring

*Patients with lesions involving posterior parietal and lateral occipitotemporal cortex are predicted to be impaired on processing words representing Observable events but should perform normally on Motionless action words. In contrast, patients with more anterior lesions sparing posterior parietal and lateral occipitotemporal cortex are predicted to be impaired on processing words representing Motionless actions but should perform normally on processing Observable event words. Impairments on Concrete, dynamic action can arise from either lesion location because the verb refers both to motion and action content. No prediction is made about processing verbs referring to Mental states*.

Brain imaging and behavioral studies alone provide limited information about the relationship between cognitive processes: motor system activation may be a consequence or correlate of comprehension, not a cause (see e.g., David and Senior, 2000 for further debate). Additional persuasive evidence comes from patients and participants with lesions affecting the brain's motor system who show a specific impairment for action knowledge; a trend that has been demonstrated for Motor Neurone Disease, Parkinson's Disease, and stroke (Neininger and Pulvermüller, 2003; Bak et al., 2006; Arevalo et al., 2007; Kalenine et al., 2010). While analogous evidence from healthy participants has been previously demonstrated in the literature with Transcranial Magnetic Stimulation (TMS), the effects found have been inconsistent (see Pulvermüller et al., 2005; Willems et al., 2010). We note here that while some participants with motor lesions do not show such deficits on action verbs (Papeo et al., 2010; Arévalo et al., 2012; Kemmerer et al., 2012; Maieron et al., 2013), none of these studies compare verbs with and without motion components, a contrast investigated as part of this study.

It has been found that visual motion features of verb meanings recruit the posterior parietal area pSTS (for reviews see Gennari, 2012; Watson et al., 2013), but also the middle temporal area of the visual cortex (known in the literature as MT/V5 or Brodmann area 19, noteworthy for its high responsiveness to visually dynamic stimuli and relatively low retinotopy; Grill-Spector and Malach, 2004). We have previously shown MT/V5 to be involved in tasks that merely imply visual motion, such as the perception of static images depicting dynamic motion (e.g., an athlete about to kick a football; Senior et al., 2002) and other studies have revealed that it is also involved during reading tasks that contain the description of motion (e.g., “the car drives toward you”; Rueschemeyer et al., 2010), with MT activation when viewing static images also mediated by the language immediately preceding it (Coventry et al., 2013). Crucially, these studies indicate that visual motion must be strongly implied in order to activate MT/V5. No studies have yet shown this for individual words nor, as noted earlier, have necessary and sufficient conditions for its involvement in the computation of language that describes motion been investigated. Further, previous work examining verbs typically confounds the semantic components of deliberate action and visual motion. Many of these studies use goal-directed actions when examining the recruitment of visual motion areas, and do not disentangle action from motion. Therefore, recruitment of visual motion areas may be contingent upon the verb containing an additional goal-directed action component.

Lateral occipitotemporal cortex (which includes MT) is associated with patterns of motion, motion related artifacts such as tools and depictions of hands (Bracci et al., 2010, 2012) and verbal material referring to actions symbolically (for a review see Lingnau and Downing, 2015). Bedny et al. (2008) are generally cited as having shown that the activation in lateral occipitotemporal cortex associated with verbs is not due to visual motion or motor activity. That fMRI study by Bedny and colleagues contrasted high-motion verbs (concrete dynamic actions such as “jump”), intermediate-motion verbs (change-of-state and bodily function) and low-motion verbs (states), showing that the amount of motion did not modulate activation of the lateral occipitotemporal cortex. However their low-motion verbs were states such as mental states and did not refer to agentive motionless actions such as “holding” or “clutching” which may indeed activate regions much more anterior to lateral occipitotemporal cortex (Kemmerer et al., 2012). Furthermore, high-motion verbs in the study by Bedny and colleagues were confounded with action, while a neater confirmation of motion associated verb activation in lateral occipitotemporal cortex would be verbs involving visual motion but not deliberate action i.e., observable events such as “crumbling” or “flowing.” In a later fMRI study by Peelen et al. (2012) showing that lateral occipitotemporal cortex is activated by state verbs (including mental states) and event verbs, the event verb category did not refer to observable events but again included concrete dynamic actions such as walking and running. Unlike previous studies, the current study delineates the action and motion element completely. The behavioral performance of patients who have sustained lesions in the left hemisphere is uniquely placed to inform our understanding of language processing by addressing this central issue.

Although lesion studies are not suitable to investigate discrete areas such as MT or pSTS, if we can show that defective motion processing is selectively associated with the posterior part of the brain housing MT and pSTS such as Brodmann area 19 or area 39, in contrast to the more anterior brain sparing those regions, we can infer that neuro-anatomically dissociable regions are activated when processing action or motion verbs, and that recruitment of these regions is necessary to derive meaning when processing modality-specific semantic knowledge. A second issue with respect to the possible links between language and recruitment of distinct neural correlates concerns the nature of the tasks used to test these links. “Levels of processing” (Craik and Tulving, 1975) refers to the degree to which a participant recruits semantic knowledge; it constitutes the qualitative difference between, for example, counting the vowels in “sinking” and knowing that “sinking” and “plunging” are more similar than “flowing” and “plunging.” Reviews (Taylor and Zwaan, 2009, 2013; Tomasino and Rumiati, 2013) find that the type of language task is a critical factor when determining the recruitment of specific brain regions. For example, semantic decisions (“Is GRASP an action?”) affect hand movements while lexical decisions (“Is GRASP a word?”) typically do not. This difference in the recruitment of alternative neural networks as a function of task requirements accounts for discrepancies within both behavioral (Lindemann et al., 2006; Sato et al., 2008) and neuroimaging paradigms (Kemmerer et al., 2008; Postle et al., 2008). In each case, a lexical, word-based decision does not result in activation of dissociable processes while a more cognitively demanding semantic task does suggesting that recruitment of neuro-anatomically dissociable regions is only necessary when recruiting semantic representations but not when making lexical decisions that do not rely on semantic information.

In our current design we accounted for these two critical issues by using tasks varying in semantic demand and words that entirely delineate the action and motion element. Firstly, to account for discrepancies in the data regarding recruitment of specific brain regions we included three tasks with different levels of cognitive demand. Our critical Semantic Similarity Judgement Task (SSJT) was expected to indicate any dissociation in action/motion verb processing in patients; as the most cognitively demanding semantic task it was considered most sensitive in identifying these dissociations. An additional Verb-Picture Matching (VPM) task was administered; easier than the SSJT but also reliant on semantic processing it was included to support the SSJT in cases of more severe stroke. Both the SSJT and VPM do not present words in isolation, but instead require comparisons to be made between two verb stimuli. A final Lexical Decision task required classification of a linguistic stimulus as a word, and was expected to rely on inherently more superficial processes that would not require the activation of dissociable processes.

Secondly, we delineated the action and motion element completely (see Table 1). As highlighted above verb content varies with some describing action (hitting), some not (desiring) while others describe motion (falling) and others not (holding). In the current fully factorial design, four verb types were used to assess the behavioral and neural independence of action and motion word processing. Verbs contained elements of action and motion (concrete, dynamic actions; “throwing”), action without motion (motionless actions; “holding”), motion without action (observable events; “flowing”), and neither action or motion (mental states; “hoping”). In doing so, the necessity of dissociable and neuro-anatomically separate regions during action and/or motion processing can be wholly explored.

Whilst the current study is not well placed to assess the critical role of the specific brain regions required when processing particular verbs due to diffuse lesion patterns and a sample size that does not allow voxel based lesion analysis, it can certainly confirm the importance of neural correlates. It is predicted that distinctive brain areas are recruited most reliably when a person accesses the relevant semantic dimension. If recruitment of additional brain areas is necessary when representing concepts, then damage to these areas may result in impaired processing of action and/or motion verbs. It is furthermore predicted that the expected dissociations will be evident in the more cognitively demanding semantic tasks but not in a lexical decision task. Finally, although included to maintain a fully factorial design, we do not make predictions about the performance of patients when processing mental state verbs, as these do not include an action or motion element.

## Materials and methods

### Participants and lesion location

#### Patients

For this multiple single-case study patients were recruited from UK National Health Hospitals/Stroke rehabilitation units located in the North East of England. Hospital admissions were screened to select patients with CT evidence of a recent ischaemic infarct or haemorrhagic stroke involving the left hemisphere. Anyone with cognitive impairment (identified from hospital screening procedures e.g., Mini Mental State Examination; MMSE), known dementia, or reported substance abuse were excluded. Patients for whom significant comprehension problems were noted in the hospital notes by clinicians or speech and language therapists beyond the acute phase of stroke were not approached because they would not cope with the tasks in this study. At test, language comprehension was further evaluated through use of the Token Task and Mississippi Aphasia Screening Test (MAST) to ensure patients could complete the experimental tasks. These tests are described below in the Screening and Patient Documentation section baed on these. Based on this criteria 25 participants were initially recruited as in-patients however 17 participants could not be followed up after discharge or did not complete all of the experimental tasks of this study.

Finally, based on the radiologist's clinical CT or MRI report we identified patients with lesions implicating either the anterior or posterior portion of the left hemisphere. Using scan images we could reliably classify six out of 8 patients. One patient was excluded because he had lacunar infarct to the left internal capsule that did not fit either anterior or posterior pattern. A second patient (patient CC) had some early signs of left hemisphere low attenuation in an otherwise nonspecific scan not allowing for classification or later lesion analysis. She had furthermore no behavioral deficits indicating a particular lesion site. She was included in the testing nevertheless as an unclassified patient and her normal performance across the experimental tasks is documented in **Table 3**. Thus the individual results of six left hemisphere patients are reported in detail in this study (3 Female, age range 52–75 years, mean 68 years 10 months, SD = 8 years 6 months,). Patients were seen at a mean time of 45.71 days (SD 13.97) post stroke. All were able to provide informed consent.

**Figure 1:**
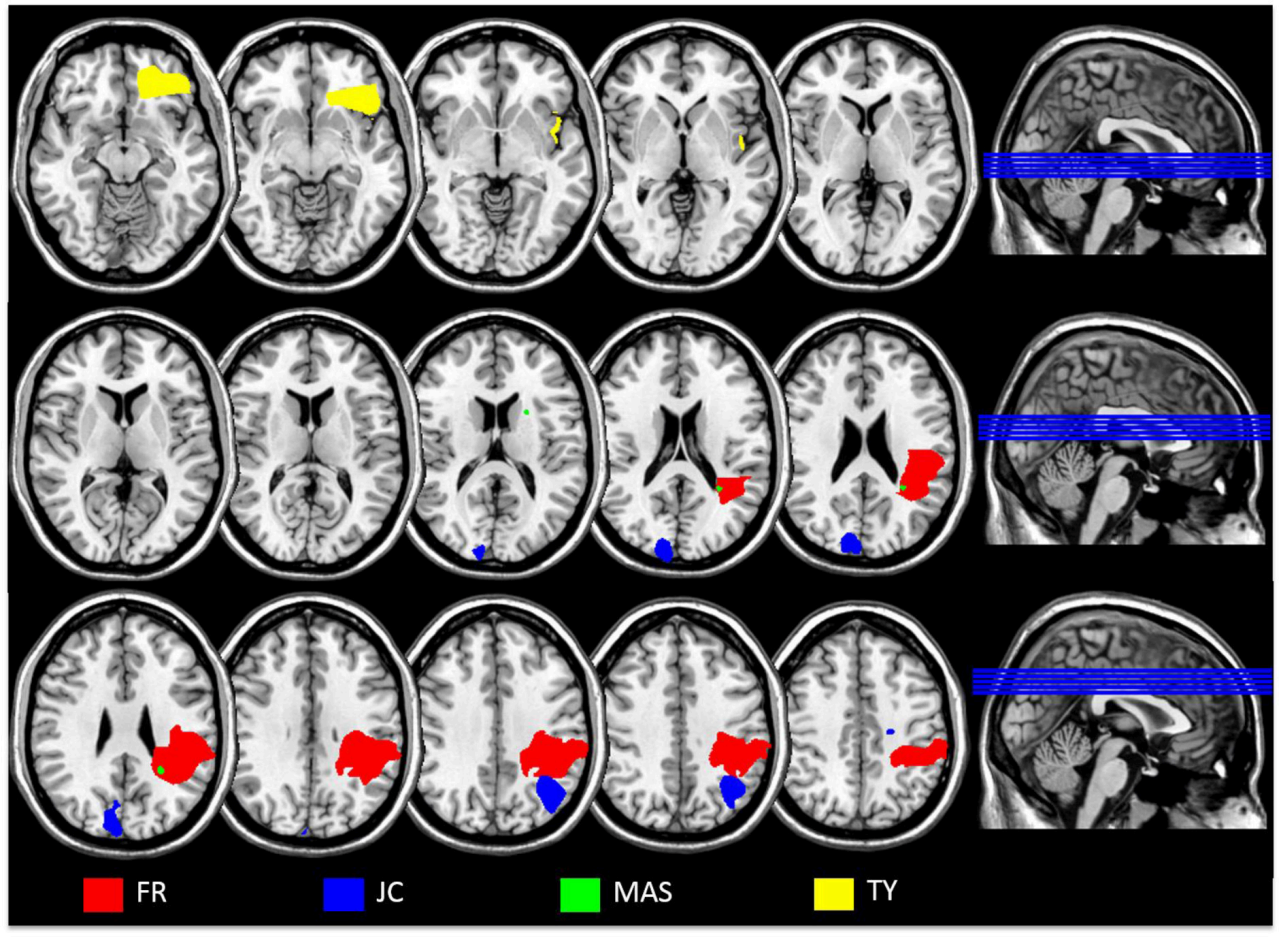
**Overlaid scan slices of each patient applied to a template scan to allow clear visualization of the anatomical landmarks using MRIcron software package (Rorden et al., 2007; http://www.mccauslandcenter.sc.edu/mricro/)**. Clinical scans could not be obtained for patient SB; the scan for DF was performed too early for the lesion to be accurately localized. Left is right as per neurological convention.

Details of each patient's lesion as identified in the CT and/or MRI reports are described below. Table 2 also lists the Brodmann areas implicated in each patient. To determine which Brodmann areas were damaged, each patient's lesions were mapped onto the digital brain image on the basis of the radiologist's report using MRIcron software package (Rorden et al., 2007; http://www.mccauslandcenter.sc.edu/mricro/mricron/). Scans were normalized (using Clinical Tool box software through SPM; Rorden et al., 2012; http://www.nitrc.org/plugins/mwiki/index.php/clinicaltbx:MainPage) and applied to the Brodmann Atlas included in MRIcron. Figure 1 includes overlaid scan slides of each patient. On the basis of scan information 3 patients (patients TY, MAS, and SB) were firmly classified as having more anterior lesions sparing the posterior parietal and lateral occipitotemporal regions of interest for motion verbs. Critically, 2 patients (patients FR and JC) had lesions involving the posterior regions of interest for motion verbs. FR had infarcts involving the left internal capsule and an old left parietooccipital lesion. JC also had lesions to the parietooccipital and lateral occipitotemporal cortex. In contrast TY had a frontal infarction that was restricted to inferior frontal and orbitofrontal territory and rostral superior and middle temporal gyrus. SB had a bleed limited to the frontal lobe. Patient MAS's lesion pattern is associated with small vessel disease affecting periventricular white matter, left temporal lobe, and left internal capsule as noted in the clinical report. As such disconnection, potentially affecting the semantic network, is probable. The multiple ill-defined white matter lesions were mostly unsuitable for mapping. However a cortical anterior lesion and small non-cortical white matter posterior lesion were identified. Furthermore, based on her symptoms of motor weakness and expressive aphasia coupled with the implication of more anterior cortical areas (BA 2, 3, 4, 8, and 40) this patient for the purpose of this study was classified as an anterior patient. In relation to the research question this is justified because the lesions in this patient spared posterior parietal and lateral occipitotemporal cortex hypothesized as associated with motion comprehension. One patient (patient DH) had an extensive lesion involving both anterior and posterior parts of the left hemisphere (left frontotemporoparietal and insula) and we therefore would not expect a dissociative pattern of impairments for processing action or motion verbs in this patient. However given that DH's lesion implicated both anterior and posterior cortical areas we felt his behavior was still relevant to the hypotheses.

**Table 2:** **Documentation of each patient**.

**Patient**	**Age at test**	**Days post**	**Right sided motor weakness on admission^a^**	**Aphasia noted on admission^a^**	**Aphasia screening (MAST) expressive/ receptive (50/50)**	**Language comprehension (stage reached of Token Test)**	**Neglect/hemianopia**	**Apraxia Score (%)^b^**	**Brodmann Areas damaged on basis of clinical scan (%** = **amount lesioned)**		
									**>75%**	**25–75%**	**<25%**
TY	74	49	Yes	Yes	49/50	5	No	98		47	11, 38
MAS	75	20	Yes	Yes	26/48	5	No	85			2, 3, 4, 8, 40
SB	72	50	No	Yes	50/48	5	Left allocentric neglect	99			
DH	68	56	Yes	Yes	17/48	6	No	90			
FR	81	33	Yes	No	50/49	6	No	96	2	40, 41	4, 21, 39, 42
JC	52	55	Yes	Yes	40/48	6	Right superior quadrantanopia	93		39	6, 7, 19, 40

*Only the scan report details are included for DH because the scan was performed too early to allow accurate localization of the full extent of this large lesion. The scan of SB could not be obtained for mapping but the radiologist's original report noting a frontal bleed leaves little uncertainty*.

a *Symptoms noted on admission were on the basis of hospital notes written by clinicians and therapists*.

b *Apraxia score (%) refers to overall accuracy across apraxia screening tests: imitation (hand and finger gestures; pathological score ≤ 17/20 on either task) and object-use tasks (pantomime and actual use; pathological score ≤ 43/53 and ≤ 16/18 respectively) with 100% meaning no errors were made on any of the tests*.

#### Healthy controls

A control group of 15 healthy older adults aged 63–84 years (mean 71 years 8 months, *SD* 6 years 2 months, 9 female) were recruited from a database of older adults held in the Department of Psychology, Northumbria University. Control participants were right handed (as were patients), and had not sustained any form of stroke or other form of brain damage. The control group received £3.00 for their participation. All procedures were approved by the local Ethics Committee within the Department of Psychology, Northumbria University as well as NHS research ethics.

### Method and procedure

Verb content varies—some involve action (hitting), some not (desiring) and some involve movement (falling), some not (holding). Because of their versatility, verbs afford firm control over semantic content and linguistic factors while tapping into different, but experimentally predictable, resources (see Table 1). The design of the current study allows an investigation of the neural systems to be involved in language comprehension. This pushes for novelty in two ways: By investigating across semantic dimensions and levels of processing.

In line with the depth-contingent processing hypothesis outlined in the introduction, we predict that non-dedicated brain areas are recruited most reliably when a person accesses the relevant semantic dimensions. Hence, anterior lesions will consistently interfere with semantic decisions on verbs describing motionless actions (A+/M−) and posterior lesions will interfere with semantic decisions on verbs describing observable events (A−/M+) only. Crucially, the more cognitively demanding semantic tasks outlined below (Semantic Similarity Judgment Task and Verb-Picture Matching; SSJT and VPM, respectively) do not present words in isolation, but in more meaningful contexts requiring comparisons to be made between stimuli; further, lexical decision merely requires classification of a linguistic stimulus as a word, while the semantic tasks require comparison. Each of these changes enhances the depth of semantic processing. We therefore predict effects in the more cognitively demanding tasks (SSJT and VPM), which rely more heavily on semantic processing, and not in the less cognitively demanding task, which relies on inherently more superficial processes. Further, we expect the SSJT to be more sensitive at identifying dissociations in verb processing (due to recruitment of non-dedicated brain regions) as it is more cognitively demanding than the VPM. In more severe stroke however, we expect the VPM to add insight into SSJT performance.

#### Screening and patient documentation

##### Mississippi aphasia screening test (MAST)

As the participants had suffered damage to the left hemisphere, language and communication skills were assessed using the Mississippi Aphasia Screening Test (MAST; Nakase-Thompson, 2004). The MAST contains nine subtests ranging from 1 to 10 items and provides indices of receptive and expressive aphasia. There was a maximum score of 50 points for each of the receptive and expressive aphasia indices which are noted for each of the patients in Table 2.

##### The token test

The general severity of any receptive aphasia was also assessed using the short version of the token test for language comprehension (De Renzi and Faglioni, 1978). As indicated in Table 2, all patients successfully followed commands consisting of at least five stages.

##### Symptoms of apraxia and neglect

A standard battery of apraxia screening tests was administered to document symptoms of apraxia. These included imitation of hand and finger gestures (Goldenberg, 1996), whereby the patient was required to copy a series of gestures that were demonstrated by the experimenter (pathological score ≤ 17/20 on either task), and pantomime (Goldenberg et al., 2007) and actual use (De Renzi and Lucchelli, 1988) of common objects (pathological score ≤ 43/53 and ≤ 16/18 for respective tasks); the examiner named the object-use action and patients were marked on the presence or absence of predefined movement features. Based on the overall performance accuracy across all apraxic screening tests, the severity of apraxia was calculated. All patients were no less than 90 percent accurate across the screen except for patient MAS who was 85% accurate. Errors in patient MAS's performance was apparent during the imitation of hand gestures (scoring 17/20) and in the form of body-part-as-object errors during object-use pantomime (scoring 31/53). Pathological scores were also noted for FR during the imitation of finger gestures (17/20) and DH during hand gesture imitation (15/20). Remaining patients did not obtain a pathological score during apraxia screening. Visuospatial neglect was assessed using the Apples Test (Bickerton et al., 2011) and is reported in Table 2. All the above standard neuropsychological tests were examined within days of the experimental assessment.

##### Object recognition screening task

Word stimuli were presented in preparation for the experimental session to establish that basic processing of written words and pictures were intact. For this task, participants were presented with a written one-word exemplar (uppercase, Arial font, size 72) and asked to read but not verbalize or attempt to verbalize the presented word. When the participant confirmed they had read the word, they were presented with the pictorial representation of the word amongst three distractors that belonged to the same semantic category. For example, circle (target), rectangle, triangle, and square (distractors). Participants had to identify which one of the four images they believed was a representation of the target word. This procedure was followed for four targets from different semantic categories: an animal (rabbit), fruit (lemon), object (clock), and shape (circle). The pictorial target and distractor stimuli for each semantic category were printed in color onto one A4 laminated sheet. The four exemplars of the aforementioned semantic categories were selected from the Snodgrass and Vanderwart (1980) set of images. None of the patients had difficulty with either of these screening tasks.

#### Experimental tasks

##### Word stimuli used in the lexical task and semantic similarity judgement task (SSJT)

Common English words (between 4 and 7 letters in length) were selected and the suffix “ing” added to disambiguate all words as verbs. Each word was allocated to one of the four conditions (see Table 1). Four independent assessors were provided with all verbs and the operationalized definitions of each condition, and rated whether they agreed (Yes/No response) to each verb/condition pairing. Only the verbs that reached a majority agreement by at least three of the four assessors were retained. A Google search of hits for each verb was used to obtain the frequency of use in the English language. Selected items were matched for letter length, number of syllables, and frequency (details are given in Appendix A).

In addition to the use of independent assessors, we also examined available linguistic resources to extract information regarding imageability and concreteness for individual verbs (Wilson, 1988; Bird et al., 2001), and existing classifications of verbs where relevant (e.g., Levin, 1993). From these resources we constructed a more limited list of verbs for final analysis: the full list and the reduced list are in Appendix (A). The reported analyses are based on the items in bold only. Of course the word lists are supposed to differ in their ratings on some of these dimensions (e.g., a +action verb is clearly more concrete and imageable than a −action verb).

To construct the stimuli for the SSJT—a task successfully implemented in previous research both in neuroimaging and clinical populations (Kemmerer et al., 2008; Fernandino et al., 2013) - each word from the final list, referred to as the “*pivot,”* was matched with a word of similar meaning (*target*), and a *distractor* word. Both the *target* and *distractor* were taken from the same semantic category as the *pivot*. Note that distractors are consistently, but only moderately, different from pivots and targets; this requires participants to think carefully about subtleties in the meanings of all three words in order to successfully complete the task. An additional four independent raters confirmed that the *target/pivot* items were more similar in meaning compared with the *distractor/pivot* items (see Appendix B for an exhaustive list of pivots, targets, and distractors).

##### Non-word stimuli used in the lexical task

A list of 52 non-words was obtained from the ARC Non-word Database (http://www.maccs.mq.edu.au/~nwdb/nwdb.html). These followed the same letter-length criteria as the word stimuli and were converted into verbs as described above. Thirteen non-words were allocated to each of the four conditions, and matched with the corresponding UK English verbs for letter-length and number of syllables. Each non-word was novel with no repetitions across the four categories (see Appendix C).

##### Picture stimuli used in the verb-picture matching task (VPM)

Two pictorial representations of each of the 52 English verbs used for the word stimuli were created. A search on Google Images identified photographic representations of each verb. An additional four independent assessors rated how closely each image represented its associated verb. An image was allocated as the *target* pictorial representation of each verb if a majority agreement of 1st choice was reached by at least three of the four assessors. The 52 images rated as 2nd choice were retained as *distractor* images. Each of the 52 *target* images were randomly paired with a *distractor* image from the same condition (i.e., the four conditions outlined in Table 1).

#### Procedure

All participants provided written informed consent and were tested either in hospital/rehabilitation unit, or at their own homes or university premises if they were healthy controls. Testing was completed over two or three sessions depending on how many tasks the participant could complete at each visit. All tasks were administered in a fixed order as below. The computerized tasks were presented to the participants using a Toshiba laptop with a 12 inch screen, and programmed using Eprime2. Participants were asked to identify the target by either stating this verbally or pointing to their choice. The participants' response was recorded by the experimenter using either a left or right mouse click. A 4-trial practice session was administered to ensure the participants understood the task instructions. If necessary this was repeated until the participant demonstrated they fully understood the task requirements. There was no maximum time limit and each set of stimuli was interspersed by a blank screen of no fixed duration to enable the participants to have a rest at any time they needed.

##### Lexical decision task

**Figure 2:**
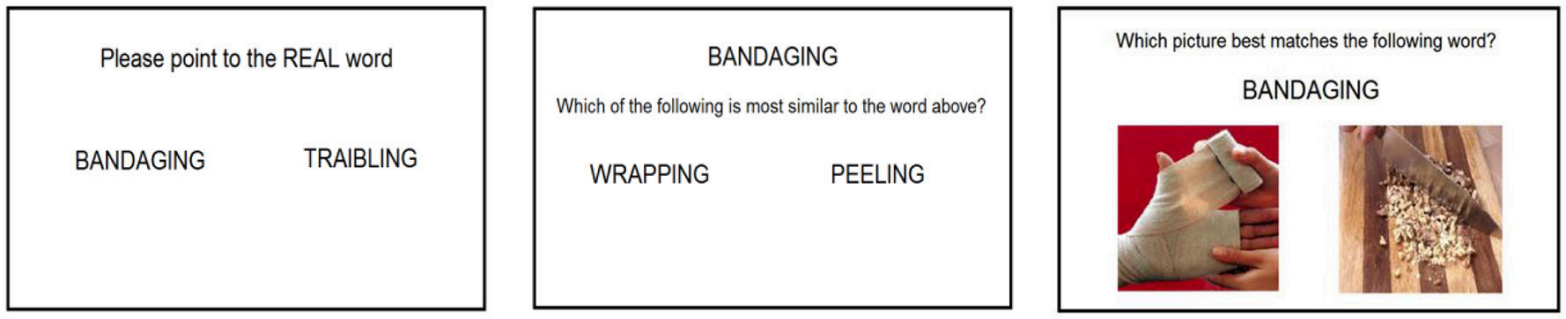
**Screen layouts (from left to right) for the lexical decision task, semantic similarity judgment task, and the verb-picture matching task**.

The participants were presented with two words on screen; one real word and one non-word. They were asked to identify which was the real word. This task is illustrated in Figure 2. Control participants were not assessed on this basic task.

##### Semantic similarity judgement task (SSJT)

The participants were advised that they would see one word in red colored text (*pivot*) at the top of the screen. Underneath they would see two words (*target and distractor*) in black text. They were instructed to choose which one of the two words in black text was most similar to the word in red. Instructions stating “*Which of the two words below is most similar to the word above”* were also presented on screen below the *pivot*. The pivot word was presented centrally in the upper third of the screen. The target and distractor words were presented centrally (vertically) and equidistant (horizontally) from the center of the screen (see Figure 2). The presentation of the target word on the left/right of the screen was counterbalanced across all trials.

##### Verb-picture matching task (VPM)

The stimuli consisted of one *pivot* word (as described in the word stimuli section) and the two pictorial representations (one *target* and one *distractor* as described in the picture stimuli section). The *pivot* word was presented centrally in the upper third of the screen. The *target* and *distractor* images were presented centrally (vertically) and equidistant (horizontally) from the center of the screen. As above the participants were advised that they would see one word in black colored text at the top of the screen. Underneath they would see two images. They were instructed to identify which one of the two images was most similar to the word above. Instructions stating “*Which picture best matches the following word”* were also presented on screen above the *pivot*. Order of presentation of the target on the left and right of the screen was counterbalanced.

### Data analysis

The data from 6 patients were included in the analyses. In order to explore the variance in individuals' performance in greater depth, a multiple single-case approach was adopted. The patients' task performance on the experimental tasks was compared to that of the healthy control group using modified *t*-tests (Crawford and Garthwaite, 2002), a standard statistical analysis which enables significance testing of individual scores compared with a control group. This method has been shown to be robust when comparing single-cases to a small control sample even in instances where such a sample is not normally distributed (Crawford et al., 2006). All patients completed the lexical, SSJT, and VPM tasks and where possible patients were retested on the critical SSJT task to confirm the pattern of results; whilst the VPM was useful for adding clarity to noisy data in cases of severe stroke, the more cognitively demanding SSJT was believed to be most reliant on the activation of semantic processes when making action/motion decisions. Retest took place 3 months after initial testing on the task (on average across patients retest took place 14 weeks and 3 days after initial testing). It was not possible to retest two of the 6 patients (patient MAS and SB) as they were not reachable after discharge. The scores on SSJT in Table 3 are those at first testing, and any changes at retest are accounted for in text where available for individual patients.

**Table 3:** **Patient percentage correct for the semantic tasks on the SSJT at initial testing, the VPM, and the Lexical Decision task**.

**Patient ***(lesion)*****	**SSJT**				**Verb-picture matching**				**Lexical**			
	**+A+M**	**+A−M**	**−A+M**	**−A−M**	**+A+M**	**+A−M**	**−A+M**	**−A−M**	**+A+M**	**+A−M**	**−A+M**	**−A+M**
CC^a^	92	100	92	100	100	100	100	100	100	92	100	100
TY^b^	100^*^^,^ ^1^	67^**^	100	67^**^	100	100	100	80	100	100	100	100
MAS^b^	89	67^**^	100	100	100	100	100	80	89	100	100	100
SB^b^	78^*^	83^*^	100	100	78^**^	83^**^	100	60^**^	89	100	100	83
DH^d^	33^**^	67^**^	17^**^	83^*^	100	83^**^	83^**^	80	100	100	100	83
FR^c^	89	100	83^*^	67^**^	100	100	100	80	100	100	100	100
JC^c^	78^*^	100	50^**^	83^*^	100	75^**^	100	50^**^	89	100	100	100
Controls (SD)	88(5)	99(4)	97(7)	97(7)	100(0)	100(0)	100(0)	88(12)	n.t.	n.t.	n.t.	n.t.

*Dark shaded areas in the table highlight the expected pattern of impairments, and light shaded areas highlight the expected dissociating intact performance*.

* p < 0.05;

** p < 0.001;

1 *Patient performance better than control group*.

a *Unclassified lesion (patient scan too early to identify lesion)*.

b *More anterior lesions sparing posterior parietal and lateral occipitotemporal cortex*.

c *Lesions involving posterior parietal and/or lateral occipitotemporal cortex*.

d *Widespread left hemisphere lesion including both posterior and more anterior regions of interest*.

## Results

Overall, the patients demonstrated dissociable deficits for action or motion verbs depending on lesion location. Inspection of the combined averaged percentage correct from initial and retest of the SSJT task (see individual results for details of duration between test/retest) for each condition identified patients with more anterior lesions sparing posterior parietal and lateral occipitotemporal cortex (TY, MAS, SB) making more errors in the motionless action (+Action/−Motion) condition (*t* = −3.631, *p* = 0.001) whilst the patients with lesions involving posterior parietal or lateral occipitotemporal cortex (FR & JC) made significantly more errors in the observable event (−Action/+Motion) condition (*t* = −3.631, *p* = 0.001).

To explore a dissociation of semantic representations for action and motion specific verbs, differences in performance on the semantic tasks (SSJT & VPM) were compared between individual patient scores and the normative data from the healthy control participants (see Table 3). The performance of patients classed as having anterior lesions are initially discussed followed by those classed as having posterior lesions.

Analysis of the results from initial testing of the Semantic Similarity Judgement Task (SSJT) confirmed that patients with more anterior lesions sparing posterior parietal and lateral occipitotemporal cortex (TY, MAS, SB) showed significantly impaired performance in the motionless action (+Action/−Motion) condition compared to control participants, suggesting a deficit in action comprehension, while performing normally on the observable event (−Action/+Motion) condition. Individual patient performance is as follows:

### Patient TY

#### Expect impaired processing of action verbs

##### Lesion and deficits

TY had a frontal infarct implicating BA 47, 11, and 38; presented with aphasia and motor weakness on admission; at test he had no symptoms of expressive or receptive aphasia and no symptoms of visual neglect or apraxia.

##### SSJT

A robust deficit was observed for processing motionless action (+Action/−Motion) items of the SSJT task at initial and retest (11 weeks, 4 days later) when compared to the control group (both *t* = −7.746, *p* < 0.001). TY was significantly impaired in the mental states (−Action/−Motion) category compared to control participants in both SSJT testing sessions (both *t* = −4.150, *p* < 0.001). TY performed at ceiling on the observable event (−Action/+Motion) condition at initial testing (*t* = 0.415, *p* = 0.342). TY was also unimpaired in the +Action/+Motion condition, performing better than controls on both test and retest sessions in this condition (both *t* = 2.324, *p* = 0.018). Of note, at retest TY's performance was impaired in the −Action/+Motion condition *(t* = −7.746, *p* < 0.001). This is difficult to interpret, but is not considered indicative of a motion processing impairment given his perfect performance in this condition in the VPM and at initial SSJT test.

##### VPM

TY's performance was at ceiling for the two critical conditions (+Action/−Motion and −Action/+Motion) as well as on +Action/+Motion (*t* = 0.00, *p* = ns) and comparable to controls on the mental state (−Action/−Motion) condition (*t* = −0.645, *p* = ns).

##### Interpretation

Performance at ceiling during the VPM does not allow interpretation, but based on SSJT performance it can be concluded that TY's performance on the initial and retest of the SSJT suggest a robust deficit specific to motionless actions (+Action/−Motion), in keeping with what was predicted on the basis of this patients frontal lobe infarction, sparing posterior parietal and lateral occipitotemporal cortex associated with motion comprehension.

### Patient MAS

#### Expect impaired processing of action verbs

##### Lesion and deficits

Lesion implicated periventricular white matter, left temporal lobe, left internal capsule (BA 2, 3, 4, 8, 40); presented with aphasia and motor weakness on admission; at test she had no symptoms of neglect but demonstrated expressive aphasia and mild apraxic symptoms.

##### SSJT

Compared to controls, MAS showed a distinct impairment in the motionless action (+Action/−Motion) condition: *t* = −7.746, *p* < 0.001; performance on remaining verb conditions were comparable to controls (see Table 3). Patient MAS' performance was at ceiling on the observable event (−Action/+Motion) condition: *t* = 0.415, *p* = 0.342, and mental state (−Action/−Motion) condition: *t* = 0.415 *p* = 0.342, and comparable to controls in the concrete, dynamic action (+Action/+Motion) condition: *t* = 0.194, *p* = 0.425.

##### VPM

MAS' performance was at ceiling for the two critical conditions (+Action/−Motion and −Action/+Motion) as well as on the concrete, dynamic action (+Action/+Motion, *t* = 0.00, *p* = ns) and comparable to controls on the mental state condition (−Action/−Motion *t* = −0.645, *p* = ns).

##### Interpretation

In conclusion, based on highly selective impairment in the critical motionless action condition of the SSJT task this patient's performance, like the above patient, is in keeping with what was predicted on the basis of this patient's more anterior lesion. Based on her post-stroke behavioral impairments and her lesion data, it is possible that disconnection, potentially affecting the semantic network, has occurred in this patient. Posterior parietal and lateral occipitotemporal cortex associated with motion comprehension are however spared.

### Patient SB

#### Expect impaired processing of action verbs

##### Lesion and deficits

SB had a frontal bleed; aphasia was observed on admission, with no symptoms of motor weakness; at test, SB showed no symptoms of aphasia or apraxia but demonstrated left allocentric neglect.

##### SSJT

SB performed poorly in the critical motionless action (+Action/−Motion) condition (t = −3.873, *p* = 0.001). Performance in the concrete, dynamic action (+Action/+Motion) condition was also lower than controls (*t* = −1.936, *p* = 0.037). Performance was comparable to controls in the observable event (−Action/+Motion) condition (*t* = 0.415, *p* = 0.342). There was no difference between SB and the control group's performance in the mental state (−Action/−Motion) condition (*t* = 0.415, *p* = 0.342).

##### VPM

Consistent with the SSJT, SB performed worse than controls in the motionless action (+Action/−Motion) condition (*t* = −16.460, *p* < 0.001) and the concrete, dynamic action (+Action/+Motion) condition (*t* = −21.301-14.254, *p* < 0.001). Unlike the SSJT, SB was significantly impaired in the mental state (−Action/−Motion) condition (*t* = −2.259, *p* = 0.002). Performance was comparable to controls in the observable event (−Action/+Motion) condition (*t* = 0.00, *p* = ns).

##### Interpretation

Although SB was impaired on a number of verb conditions, the dissociation between impaired motionless action (+Action/−Motion) comprehension and intact comprehension of observable events (−Action/+Motion) was clearly evident based on the combined SSJT and VPM performance in this patient. This was predicted based on the frontal bleed sparing posterior parietal and lateral occipitotemporal cortex.

### Patient DH

#### Expect impairment in processing either/both action/motion verbs

##### Lesion and deficits

DH suffered a significant stroke leaving him quite impaired; aphasia and right motor weakness were noted on admission and at test DH had severe expressive aphasia, but no visual neglect or apraxia. His clinical scan was performed very early on; too early to reliably localize the lesion. Based on the radiologist's report describing a lesion in the left fronto-temporo-parietal infarct and insula and his disfluent speech indicative of a frontal lesion, DH was classed as both anterior and posterior. It was therefore predicted that this patient would not present a neat dissociation in verb processing performance. This wide-spread damage also seems to be reflected in his non-specific behavior on the experimental tasks.

##### SSJT

DH performed poorly across this task on initial test and retest, which may be attributable to the severity of his stroke. At both initial and retest, DH was significantly less accurate across all conditions compared to the control group (all *p* ≤ 0.037). Initial testing did not reveal a clear pattern of behavior (see Table 3); DH showed the most notable deficit in the observable event (−Action/+Motion) condition (*t* = −11.066, *p* < 0.001) followed by the concrete, dynamic action (+Action/+Motion) condition (*t* = −10.651, *p* < 0.001). At retest and still significantly impaired compared to the controls, DH's performance improved in both the observable event (−Action/+Motion) and concrete, dynamic action (+Action/+Motion), but fared considerably worse in the motionless action (+Action/−Motion) condition.

##### VPM

Unlike the SSJT, DH's behavior on the less demanding VPM task showed more specific deficits. Compared to controls, DH's performance was significantly poorer in the motionless action (+Action/−Motion) condition (*t* = −16.460, *p* < 0.001) as well as on and the observable event (−Action/+Motion) condition (*t* = −16.460, *p* = < 0.001). In contrast performance was normal on concrete dynamic action (+Action/+Motion; *t* = 0.00, *p* ns) and in the mental state (−Action/−Motion; *t* = −0.645, *p* = 0.265) condition.

##### Interpretation

Although the pattern of results with this patient is somewhat clouded by a general level of impairment (i.e., performing poorly across many conditions on the more demanding SSJT task) it is interesting that this patient on the VMP was impaired only on the two critical experimental conditions, observable events associated with posterior damage and motionless actions associated with more anterior damage, while managing normal performance on the other two conditions of the VPM task, concrete dynamic action and mental states. In conclusion, this patient showed the non-selective pattern of behavior predicted by his lesion involving both areas of interest.

### Patient FR

#### Expect impairment in processing motion verbs

##### Lesion and deficits

Lesion implicated the left internal capsule and left parieto-occipital region (BA 40, 41 4, 21, 39, 42); aphasia on admission without right motor weakness; at test FR had no symptoms of aphasia, neglect, or apraxia.

##### SSJT

FR showed poor performance in the critical observable event (−Action/+Motion) condition at initial test (*t* = −1.936, *p* = 0.037) and retest (*t* = −4.150, *p* < 0.001) 21 weeks 6 days later, suggesting a robust motion deficit (see Table 3). Performance on the mental state (−Action/−Motion) condition at initial testing (*t* = −4.150, *p* < 0.001) and retest (*t* = −1.936, *p* = 0.037) was significantly poorer than controls. Normal performance was however observed in the motionless action (+Action/−Motion; *t* = 0.242, *p* = 0.406) and the concrete, dynamic action (+Action/+Motion; *t* = 0.194, *p* = 0.425) conditions compared with controls.

##### VPM

FR's performance was comparable to controls across conditions (all *p* ≥ 0.265), performing largely at ceiling. This may be indicative of his mild stroke.

##### Interpretation

A distinct –Action/+Motion deficit with maintained +Action/−Motion and +Action/+Motion performance in the SSJT suggests that FR presented with an isolated deficit in the comprehension of motion verbs in line with a lesion involving posterior parietal cortex.

### Patient JC

#### Expect impairment in processing motion verbs

##### Lesion and deficits

Parieto-occipital infarct implicating BA 39, 6, 7, 19, 40; aphasia, right motor weakness and right superior quadrantanopia on admission; at test showed mild expressive aphasia but no symptoms of apraxia.

##### SSJT

JC demonstrated a reliable motion deficit for observable event (−Action/+Motion) at initial test (*t* = −6.501, *p* < 0.001) and retest (*t* = −4.150, *p* < 0.001) 11 weeks 4 days later. Impaired performance was also observed at initial test and retest in the concrete dynamic action (+Action/+Motion): both *t* = −1.936, *p* = 0.037, and mental state (−Action/−Motion) condition: both *t* = −1.936, *p* = 0.037. JC's performance was equivalent to the control participants at both the initial test and retest in the motionless action (+Action/−Motion) condition (both *t* = 0.242, *p* = 0.406).

##### VPM

Unlike SSJT, JC performed significantly worse in both the motionless action (+Action/−Motion; *t* = −24.206, *p* < 0.001) and mental state (−Action/−Motion; *t* = −3.066, *p* = 0.004) conditions compared with the control group. Performance was comparable to controls for the dynamic action (+Action/+Motion) and observable event (−Action/+Motion) conditions (both *t* = 0.00, *p* = ns).

##### Interpretation

Although the contrast between this patient's performance on the SSJT and VPM tasks introduces an element of uncertainty, it is worth noting that performance on the VPM task was not reflected in other tasks. On the basis of the SSJT task performance at both initial test and retest this patient presented with a dissociation between impaired comprehension of motion associated observable events and intact comprehension of motionless actions, in line with this patient's lesion involving both posterior parietal cortex and lateral occipitotemporal cortex.

##### Lexical decision task

As predicted, the pattern of dissociations was evident on the semantic task, but not the lexical processing task. Patients performed worse than the healthy control participants in the semantic tasks and these deficits were selective across the action present/motion present conditions. Conversely, patients performed accurately in the lexical decision tasks and showed hit rates substantially higher compared to hit rates in the semantic tasks, with patients performing at ceiling or making very few errors (see Table 3).

To summarize the pattern of dissociations, patients with more anterior lesions sparing posterior parietal cortex and lateral occipitotemporal cortex (TY, MAS, and SB) were consistently poorer on tasks involving verbs describing motionless actions (+Action/−Motion). On the other hand, patients with lesions involving posterior parietal cortex and lateral occipitotemporal cortex (FR, JC) were consistently poorer on tasks involving verbs describing observable events (−Action/+Motion), while patient DH with a large lesion involving both areas of interest did not show dissociate behavior.

## Discussion

In conditions where verbs contained action and/or motion content, patients with lesions involving posterior parietal and lateral occiptotemporal cortex show a selective deficit on semantic decisions regarding verbs that afford motion. Patients with lesions sparing these posterior regions associated with motion processing showed the opposite pattern of selective deficits in action verb processing but intact motion verb processing. The dissociation between action and motion routes to verb understanding is important. In past studies verbs depicting actions have been considered primarily in relation to motor/premotor activations—but actions depict motions as well as actions. For that reason, the variable results found in past studies may partly be a function of two routes to understanding verbs—action and motion. In the patients we have found dissociations between verbs affording motion-only and verbs affording action-only in cognitively demanding semantic tasks. The opposite pattern of results was seen in patients where posterior regions associated with motion were spared: these patients performed poorly on verbs affording actions but not motion while they performed well on verbs affording action but not motion. Whilst in this small sample we cannot perform detailed lesion analyses, the fact that this selectivity is associated with specific anterior/posterior lesion patterns has implications for most assumptions about action verb understanding, indicating multiple routes to comprehension. This would be consistent with recent work on understanding goals and intentions through actions, with evidence that motor/premotor system activation might be one of several routes to action understanding (Eshuis et al., 2009; Gredebäck and Melinder, 2010).

Most broadly, these results contribute to our understanding of language processing as an integrated phenomenon that involves the contribution of knowledge representation from a wide variety of sensorimotor modalities (Barsalou, 1999; Taylor and Zwaan, 2008), converging with the perspective (Binder and Desai, 2011; Yee et al., 2013) that semantic knowledge is distributed across brain areas corresponding to the sensory-functional and sensorimotor characteristics of the referent. In this respect, our findings converge with findings from a variety of methodological approaches demonstrating overlapping neural substrates between language and the motor cortex, including transcranial magnetic stimulation (TMS; Buccino et al., 2005; Pulvermüller et al., 2005), magnetoencephalography (MEG; see Hauk et al., 2008 for review), fMRI (Kemmerer et al., 2012), and behavioral studies (see Glenberg et al., 2013 for a review). Our results most closely relate to those of TMS paradigms, as the temporary “artificial lesions” created in healthy participants in a TMS study are reflected in the natural lesions of our sample of participants, allowing us to draw inferences about the substantive contribution these brain areas make to semantic decisions.

All patients in the current study performed at ceiling level on the lexical decision task, which required identification of a real word against a pronounceable and equivalent non-word distractor (e.g., “praying” vs. “pibbling”). This suggests that a lexical decision does not rely on the recruitment of alternative neural networks. The predicted pattern of dissociations was evident however in the more cognitively demanding semantic tasks. The word-based SSJT task, in which required participants to decide whether “praying” was more similar to “wishing” or “judging,” was distinctly affected by the different brain lesions that were revealed by the patients studied here. To a large extent results from the picture-based VPM, which required participants to identify a picture for example of a person praying, mirrored those observed in the SSJT for verbs containing an action and/or motion content. Whilst easier than the word-based SSJT but also reliant on semantic processing, the VPM added clarity to poor performance on the SSJT. In particular, patient DH who had suffered a severe stroke, was consistently poor across conditions of the SSJT but only showed poor performance on the critical conditions of the VPM with normal performance on the neutral conditions. Together, performance across the three tasks emphasizes that recruitment of dissociable neural processes is dependent upon task requirement and cognitive demand, which may explain discrepancies found in previous data (Lindemann et al., 2006; Kemmerer et al., 2008; Postle et al., 2008; Sato et al., 2008).

It is worth noting that while the patients show statistically reliable, specific, and robust deficits in the predicted semantic categories, these selective impairments were remarkably subtle and not a reflection of typical aphasia, with receptive performance on the diagnostic screening for aphasia (MAST) near ceiling level (scoring 48 out of 50 or above) for most of our patients. Similarly, all patients performed near ceiling on the lexical decision task, with aggregate accuracy over 95%. These results promote awareness that language deficits resulting from stroke may be subtler than previously imagined, or assumed by current diagnostic material.

At the same time it should be noted that language is usually studied in cognitive psychology laboratories removed from language in the real world. Seeing the word “STOP” on a red sign at a busy traffic intersection is quite different from seeing the word STOP in black text on a white background in an experimental psychology laboratory and as such laboratory based work may lack the ecological validity required to fully understand the cognitive mechanisms that mediate natural language (e.g., Zwaan, 2009). Thus differing aspects of context, motivation, and task may result in drastically different psychological and neurophysiological responses. The choice of language task has serious implications for the identification of language problems. Cognitively demanding semantic tasks are more useful for identifying more distributed neural networks associated with language processing as lexical decisions may not require the recruitment of dissociable brain regions. Further, one of the hallmarks of language is its contextual versatility—from identification of words to conversations requiring extensive inferences and social comprehension. The latter, more semantically rich, contexts are particularly important to tap in neuropsychological testing, as exactly these tasks recruit more distributed neural networks. The current finding that specific parts of the distributed network give rise to selective impairments resonates with an emerging proposal in the cognitive sciences holding that the brain areas and networks associated with an event are a function of context, task, and strategies, not simply constrained within the domain of a particular stimulus (Tomasino and Rumiati, 2013; Bracci et al., 2016). Indeed it emerges that recruitment of several neural networks may be critical to derive meaning from language.

As predicted semantic representations for concrete, dynamic action verbs may be associated with lesions either related to action or motion processing. Indeed, we did not find the selective association with lesion location that we found for motionless events in posterior patients and observable events not associated with bodily action in patients with more anterior lesions. Perhaps more interesting, we did see impairments on processing verbs representing mental states in a number of patients who were not impaired on some of the other verb categories but as predicted without an associated lesion pattern. Although this leaves open the possibility that semantic content regarding motionless and “actionless” mental states is behaviorally and neurally independent from other verbs, this falls outside the remit of the investigation focussed on the independence of action and motion representation and its relationship to posterior parietal and lateral occipitotemporal cortex. Nevertheless, representations for verbs describing mental events in particular are left unresolved, as in previous work by Peelen et al. (2012) for example, where mental state verbs like “she believes” were mixed in with state verbs such as “she is liked,” “he lies down” or “she equates.” To what extent do verbs referring to mental states rely on visual and motor systems? Existing theories and results on this are particularly conflicted (Gallese and Lakoff, 2005; Rüschemeyer et al., 2007; Postle et al., 2008; Vigliocco et al., 2009; Dove, 2009). With regard to current results, it is worth highlighting that data coming from patients with such mixed lesion patterns do not generate results that are entirely clear cut, as is often the case with neuropsychological research.

A further inherent weakness of the current study—and potentially an area for improvement in future—concerns the selection criteria for items. First, the observable events category contains a small number of lexical items, placing an artificial constraint on the number of verbs possible in the present study. Second, natural confounds exist between verb classes; for example, observable events should inherently have higher imageability and concreteness ratings than mental events. This may also account for poor performance in verbs representing mental states in some patients. During the SSJT, four of the 6 patients performed significantly worse than controls when processing mental state verbs, which was consistent for two of these patients (SB & JC) in the Verb-Picture Matching task. Control participants also showed a drop in performance in the mental state condition of the VPM compared to other conditions. It is likely that the abstractness of these −Action/−Motion verbs, particularly in pictorial form, is generally more difficult to process, resulting in reduced performance in the mental state condition. Nevertheless, we reiterate that performance during mental state decisions cannot be used to evaluate dissociations when processing verbs involving action or motion and therefore do not discredit our other findings in the remaining stimuli. Third, only four raters assessed our categorization—and even they failed to reach a universal consensus on the full list of items. In the present study, then, we faced an inherent trade-off between statistical power and experimental validity. In future, perhaps more robust selection criteria—for example, including imageability and concreteness ratings for fewer stimuli that enjoy more universal agreement on category - might shift the balance toward improved methodological rigor at the expense of statistical power.

Establishing whether similar effects can be found in healthy participants with artificially-induced “lesions” is critical to demonstrating that these brain regions are in fact essential to action understanding in healthy populations (Taylor and Zwaan, 2009). However the current study is limited by a small sample size preventing the identification of specific non-dedicated cortical regions being determined. Further study would require a larger sample to enable voxel based lesion analyses to pinpoint the critical role of specific brain regions when processing action/motion verbs. The current results must therefore be considered within the larger context of behavioral and neuroscience research (e.g., Lingnau and Downing, 2015). Most immediately the current experimental design and hypotheses lend themselves to replication, both in other patients and in healthy participants who take part in transcranial magnetic stimulation (TMS) protocols in the way we delineated motion and action dimensions completely. Such results would bolster the claims here, showing that they are neither patient centered artifacts nor a bias of stroke victims more broadly. Note, however, that over time patients may well develop alternative routes to understanding—a point that TMS cannot speak to.

Recent advances in imaging analyses using connectivity analysis will afford investigation of the interplay between action and motion processing regions. Such interplay may allow us to explain when +Action/+Motion verbs are preserved or impaired in patients with specific lesions and furthermore reveal potential differential representation of the interesting *Mental States* verb category.

Neuroimaging work with healthy participants has identified brain activity mapping onto discrete cortical areas for action, motion, contact, and state change (Kemmerer et al., 2008). Previous neuropsychology research has demonstrated a dissociation between action verbs, which tend to be impaired by anterior lesions, and concrete nouns which are impaired by posterior lesions (Neininger and Pulvermüller, 2003). One of the key contributions of the present work is to elucidate the causality behind these effects and to demonstrate a dissociation *within* a lexical category. Future work may consider the causality of such activity and build an account of “abstract” concepts, even if this begins with an account of verbs that are not both concrete and have an immediate sensory or bodily referent.

## Author contributions

All authors were involved in the conception if this study. LT, CE, JG, and MI designed the study. CE and JG collected and analyzed the data. LT, CE, JG, and MI collaboratively drafted the manuscript and all authors approved the final version for submission.

### Conflict of interest statement

The authors declare that the research was conducted in the absence of any commercial or financial relationships that could be construed as a potential conflict of interest.
