# Supplementary material for: Dissociation between Semantic Representations for Motion and Action Verbs: Evidence from Patients with Left Hemisphere Lesions
Source: Front Hum Neurosci. 2017 Feb 14;11:35. doi: 10.3389/fnhum.2017.00035 (PMC5306207; doi:10.3389/fnhum.2017.00035)
Supplement: Supplementary file 1 [file DataSheet1.docx]

Appendices

Appendix A: Full set of word stimuli presented for each of the four Action and Motion categories of each task. Only items in bold were retained for analysis after we removed a number of items that were not deemed to be clear-cut based on available linguistic resources to extract information regarding imageability and concreteness for individual verbs (Wilson, 1988; Bird, Franklin, & Howard, 2001), and existing classifications of verbs where relevant (Levin, 1993).

|  | **Concrete, dynamic actions** | **Motionless actions** | **Observable events** | **Mental states** |
| --- | --- | --- | --- | --- |
|  | **bandaging** | attending | blooming | advising |
|  | banging | bargaining | clattering | amending |
|  | **chopping** | **clasping** | **crumbling** | appointing |
|  | **Cutting** | **clinging** | **drifting** | banishing |
|  | **digging** | **clutching** | **floating** | **desiring** |
|  | **mopping** | drooping | **flowing** | **doubting** |
|  | **rubbing** | embracing | lurching | emitting |
|  | **scratching** | **holding** | **plunging** | **hoping** |
|  | **scribbling** | lighting | printing | **liking** |
|  | squashing | loitering | **slipping** | blessing |
|  | **throwing** | **ogling** | slumping | **pondering** |
|  | tossing | **slouching** | wilting | praying |
|  | waxing | storing | yawning | **wishing** |
| Length | 7.84 | 8.23 | 8 | 7.84 |
| Syllables | 2.15 | 2.38 | 2.15 | 2.54 |
| Frequency | 70,302,000 | 87,420,300 | 86,483,385 | 74,729,000 |
| *Bold items* |  |  |  |  |
| Length | **8.11** | **7.83** | **8** | **7.33** |
| Syllables | **2.22** | **2.17** | **2.17** | **2.33** |
| Frequency | **86,176,222** | **134,243,333** | **64,316,667** | **102,316,667** |

Appendix B: Stimuli for the Semantic Similarity Judgement Task

| Concrete, dynamic action (+A+M) | | | **Motionless action (+A-M)** | | |
| --- | --- | --- | --- | --- | --- |
| Pivot word | **Target** | **Distractor** | **Pivot word** | **Target** | **Distractor** |
| bandaging | **wrapping** | **peeling** | attending | watching | Glancing |
| banging | whacking | pricking | bargaining | haggling | Buying |
| chopping | **dicing** | **scraping** | **clasping** | **clinging** | **storing** |
| cutting | **slicing** | **mashing** | **clinging** | **clutching** | **Saving** |
| digging | **shovelling** | **carving** | **clutching** | **squeezing** | **Touching** |
| mopping | **scrubbing** | **chopping** | drooping | slouching | Leaning |
| rubbing | **massaging** | **tearing** | embracing | hugging | Greeting |
| scratching | **rubbing** | **tapping** | **holding** | **gripping** | **Touching** |
| scribbling | **scrawling** | **writing** | lighting | igniting | Switching |
| squashing | smashing | flicking | loitering | waiting | Lounging |
| throwing | **tossing** | **catching** | **ogling** | **staring** | **Peeking** |
| tossing | flinging | scraping | **slouching** | **drooping** | **Tilting** |
| waxing | polishing | scrubbing | storing | saving | Switching |

| Observable event (-A+M) | | | **Mental state (-A-M)** | | |
| --- | --- | --- | --- | --- | --- |
| Pivot word | **Target** | **Distractor** | **Pivot word** | **Target** | **Distractor** |
| blooming | blossoming | sprouting | advising | suggesting | Insulting |
| clattering | rattling | rumbling | amending | changing | Doubting |
| crumbling | **breaking** | **creasing** | appointing | hiring | Arguing |
| drifting | **floating** | **lurching** | banishing | condemning | Hating |
| floating | **drifting** | **clattering** | blessing | praising | Recalling |
| flowing | **coursing** | **resting** | **desiring** | **wanting** | **Liking** |
| lurching | slumping | blooming | **doubting** | **opposing** | **Altering** |
| plunging | **sinking** | **flowing** | emitting | shining | Drifting |
| printing | copying | falling | **hoping** | **wishing** | **Enjoying** |
| slipping | **tripping** | **limping** | **liking** | **approving** | **Blessing** |
| slumping | lurching | falling | **pondering** | **thinking** | **remembering** |
| wilting | withering | crumbling | praying | wishing | Enjoying |
| yawning | snoozing | reading | **wishing** | **hoping** | **Thinking** |

Appendix C: Word / Non-word pairing for the lexical task

| Concrete, dynamic actions (+A+M) | | **Motionless actions (+A+M)** | | **Observable events (-A+M)** | | **Mental states (-A-M)** | |
| --- | --- | --- | --- | --- | --- | --- | --- |
| Target | **Distractor** | **Target** | **Distractor** | **Target** | **Distractor** | **Target** | **Distractor** |
| bandaging | **traibling** | attending | skoreling | blooming | twusting | advising | tarbling |
| banging | macting | bargaining | glickering | clattering | spromining | amending | bawthling |
| chopping | **snaiting** | **clasping** | **twafting** | **crumbling** | **knarbling** | appointing | aflurnting |
| cutting | **geebing** | **clinging** | **stedging** | **drifting** | **fanching** | banishing | vourating |
| digging | **pooting** | **clutching** | **spaicking** | **floating** | **whesping** | **desiring** | **seegling** |
| mopping | **lunting** | drooping | smatting | **flowing** | **draling** | **doubting** | **cronzing** |
| rubbing | **zeeging** | embracing | quartling | lurching | smarsing | emitting | deetling |
| scratching | **spreliching** | **holding** | **linzing** | **plunging** | **keedging** | **hoping** | **Futing** |
| scribbling | brouttling | lighting | scolting | printing | foolting | **liking** | **rebing** |
| squashing | thrudding | loitering | sleebling | **slipping** | **phurbing** | **blessing** | **skebbing** |
| throwing | **jurnging** | **ogling** | **ebling** | slumping | floosing | **pondering** | **knarbling** |
| tossing | veffing | **slouching** | **dringling** | wilting | nosting | praying | linzing |
| waxing | lejing | storing | merning | yawning | sloning | **wishing** | **touning** |
